# Tricritical point from high-field magnetoelastic and metamagnetic effects in UN

**DOI:** 10.1038/s41598-017-06154-7

**Published:** 2017-07-26

**Authors:** K. Shrestha, D. Antonio, M. Jaime, N. Harrison, D. S. Mast, D. Safarik, T. Durakiewicz, J.-C. Griveau, K. Gofryk

**Affiliations:** 1 Idaho National Laboratory, Idaho Falls, Idaho 83402 USA; 2 Los Alamos National Laboratory, Los Alamos, New Mexico 87545 USA; 3 Department of Chemistry and Biochemistry & High Pressure Science and Engineering Center, University of Nevada, Las Vegas, Nevada 89154 USA; 4 Institute of Physics, Maria Curie-Sklodowska University, 20-031 Lublin, Poland; 5 European Commission, Joint Research Centre, Directorate for Nuclear Safety and Security, Postfach 2340, D-76125 Karlsruhe, Germany

## Abstract

Uranium nitride (UN) is one of the most studied actinide materials as it is a promising fuel for the next generation of nuclear reactors. Despite large experimental and theoretical efforts, some of the fundamental questions such as degree of 5 *f*–electron localization/delocalization and its relationship to magneto-vibrational properties are not resolved yet. Here we show that the magnetostriction of UN measured in pulsed magnetic fields up to 65 T and below the Néel temperature is large and exhibits complex behavior with two transitions. While the high field anomaly is a field-induced metamagnetic-like transition and affects both magnetisation and magnetostriction, the low field anomaly does not contribute to the magnetic susceptibility. Our data suggest a change in the nature of the metamagnetic transition from first to second order-like at a tricritical point at *T*_*tri*_ ∼ 24 K and *H*_*tri*_ ∼ 52 T. The induced magnetic moment at 60 T might suggest that only one subset of magnetic moments has aligned along the field direction. Using the results obtained here we have constructed a magnetic phase diagram of UN. These studies demonstrate that dilatometry in high fields is an effective method to investigate the magneto-structural coupling in actinide materials.

## Introduction

**Figure 1:**
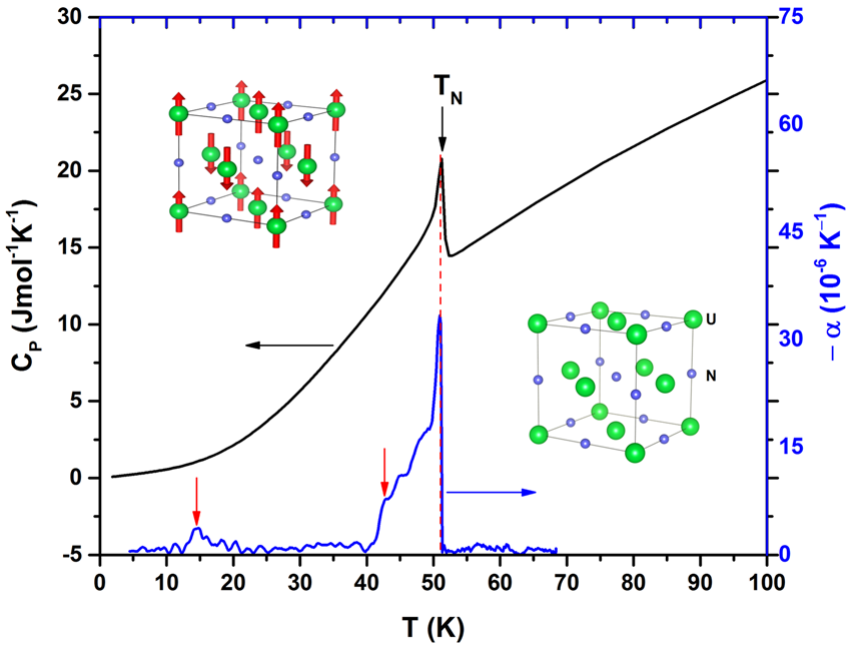
The temperature dependence of the heat capacity of UN (left hand scale) and the thermal expansion coefficient (−*α*) (right hand scale). A black arrow marks the Néel temperature at 52 K and red arrows mark anomalies in *α* described in the text. Lower inset: crystal structure of UN. Upper inset: magnetic structure of UN (see the text). Large green circles represent uranium atoms and small blue circles mark nitrogen atoms.

The development of newer and better nuclear fuel is of paramount interest for the future of nuclear power generation^1, 2^. Uranium nitride has emerged as a contender to replace uranium dioxide in alternative fuel reactors. This is due to its high thermal conductivity, high fissionable density, and higher melting temperature than other conventionally used nuclear fuels. UN crystalizes in a face center cubic (fcc) NaCl-type crystal structure with lattice parameter *a* = 4.889 *Å* ^3, 4^ (see lower inset to Fig. 1). It orders antiferromagnetically below the Néel temperature *T*_*N*_ = 52 K^5^ and neutron diffraction experiments reveal that UN forms an antiferromagnetic type-I (single-*k*) structure with an ordered magnetic moment of 0.75 *μ*_*B*_ at the uranium site^6^ (see upper inset to Fig. 1). Many experimental studies have been carried out to understand its various physical behaviors, such as electrical transport^6–8^ magnetic^9, 10^ high pressure^11, 12^ thermodynamic^13, 14^. However, despite extensive studies on UN, some of the fundamental questions of itinerant or mixed (itinerant + localised)^6, 15–19^ behavior of 5 *f* electrons and especially its interaction with magneto-vibrational properties are not clearly understood yet.

In order to better understand the magnetic and magneto-elastic properties of UN we have carried out magnetostriction (MS) and magnetisation measurements on oriented UN single crystals at low temperatures and in pulsed magnetic fields up to 65 T. Magnetostriction is a magnetic property of solids that causes a change of dimensions or shape when subjected to a magnetic field. The effect was first identified by Joule in 1842 when observing a sample of iron^20^. In general, the magnetostrictive phenomena can be classified in two types: a spontaneous magnetostriction which causes a volume change of magnetic materials without applying a magnetic field, and the forced (or linear) magnetostriction, which causes a length change when applying magnetic field. The magnetostriction is an important magnetic property and gives valuable information to solve the magnetic phase transitions, and has been successfully used to study magnetic interactions in various systems such as complex magnetic materials and textures, spin-chain compounds or/and quantum magnets^21–24^. In 5 *f*-electron systems the magnetic phase transitions mostly occur at very high magnetic fields due to strong spin-orbit coupling, and often measurements in pulsed fields are required. Van Doorn and de V. du Plessis carried out a conventional strain gauge technique to measure the lattice expansion of UN^7^. They observed a relatively large Invar type positive lattice expansion of ∼150 × 10^−6^ (150 *μ* Strain) in the magnetically ordered state. The magnetisation measurements of UN powder done by Shinkel and Troć^9^ show a linear dependence with field up to 30 T. A recent magnetisation study by Troć *et al*. extended the field range to 65 T. They observed a metamagnetic transition at high fields and proposed a magnetic phase diagram of UN^19^. Here we show that the MS of UN measured below *T*_*N*_, and along [100] and [110] crystallographic directions is large and shows complex behavior with two distinct transitions. The high field transition (∼50 T) is of metamagnetic type, most probably caused by a spin-flip of magnetic moments under applied magnetic field. This transition is consistent along the two crystallographic directions and observed in both MS and magnetisation measurements. However, the low field anomaly (∼10 T) is only observed in MS, indicating this transition causes measurable sample length change but does not affect magnetisation. The presence of hysteresis at low fields in our MS measurements might suggest a magnetic domain origin of this anomaly. Furthermore, the magnetostriction and magnetisation indicate a change in the nature of the metamagnetic transition from first to second order-like at a tricritical point at *T*_*tri*_ ∼ 24 K and *H*_*tri*_ ∼ 52 T. In addition, the magnetisation does not saturate even at magnetic fields as high as 60 T and the induced magnetic moment is almost one third of the value estimated by previous neutron scattering measurements^25^. These results have been combined to construct a new (H, T) magnetic phase diagram of UN.

## Results and Discussion

**Figure 2:**
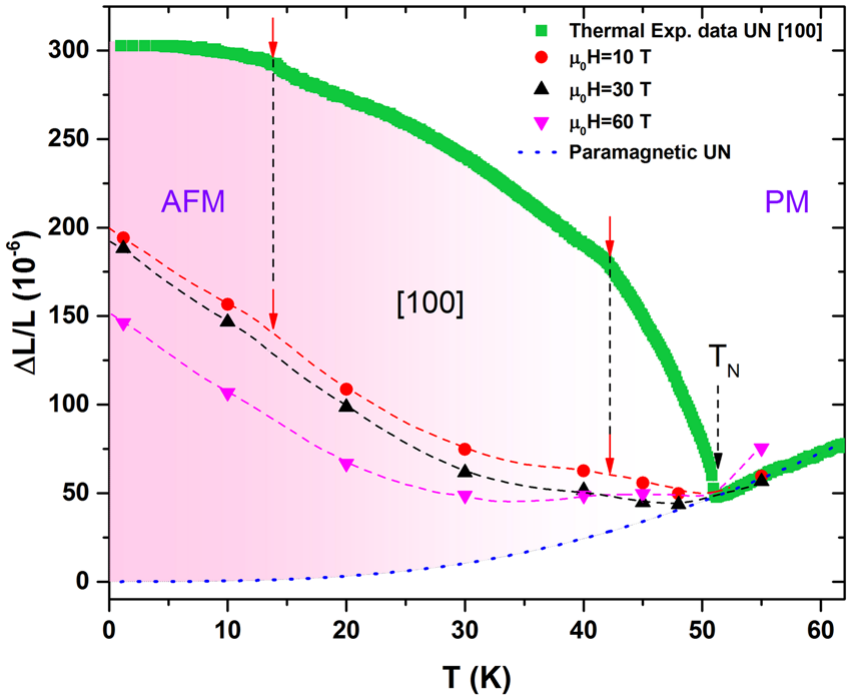
The temperature dependence of the thermal expansion of the UN measured along [100] (solid green symbols). Two anomalies at *T* = 15 and 42 K are marked by blue arrows. The blue dotted line is a hypothetical lattice behavior obtained by the Grüneisen approximation for non-magnetic UN (see text). The solid circles and triangles show the thermal expansion at magnetic fields 10, 30 and 60 T. The dashed lines act as a guide for the eye. The dashed arrows marks the magnetic phase transitions at 52 K.

Figure 1 shows the temperature dependence of heat capacity of UN. A pronounced *λ*-type anomaly observed at *T*_*N*_ = 52 K reflects the bulk antiferromagnetic property and good quality of the UN crystal. The temperature dependence of the thermal expansion coefficient −*α* (blue line), obtained by taking the first derivative of the thermal expansion curve shown in Fig. 2, is plotted on the right hand scale. As seen from the figure, *α* shows a sharp peak at *T*_*N*_ consistent with the pronounced peak in heat capacity. Moreover, there are two other anomalies around 15 and 42 K, as pointed out by the red arrows. These anomalies were not observed in the previous thermal expansion studies most probably due to the resolution of the measurements. Interestingly, the magnetic specific heat, resistivity, thermopower, and thermal conductivity measurements^6, 19, 26^ also show anomalies at the same temperatures. The origin of these transitions is not clear, however, it was proposed that the low temperature anomaly at 15 K could be due to the formation of a spin density wave state^19^.

The linear thermal expansion (TE) of oriented UN crystals was performed using a recently developed Fiber Bragg Grating (FBG) method^27^ (see Methods Section). Figure 2 shows the thermal expansion, defined as $\frac{{\rm{\Delta }}L}{L}(T)$ where Δ*L* is the sample length change with respect to the original length *L*, of the UN single crystal along the [100] direction. The overall shape of the $\frac{{\rm{\Delta }}L}{L}(T)$ curve is similar to previously observed by van Doorn and de V. du Plessis^7^. With cooling, the lattice shrinks as expected and then rapidly expands below *T*_*N*_, characteristic of Invar effect^28^.  The positive and large magnetostriction below *T*_*N*_ implies strong correlations between spin and lattice degrees of freedom in the antiferromagnetic state^28, 29^. For comparison we have also included a hypothetical lattice change of UN in the absence of antiferromagnetic ordering, as shown by the dotted blue line. This was obtained by fitting the second-order Grüneisen approximation^30, 31^ to the $\frac{{\rm{\Delta }}L}{L}(T)$ of UN in the paramagnetic state using the Debye temperature Θ_*D*_ = 324 K^13^. The difference between the extrapolated Grüneisen dependence in the magnetically ordered region and the measured thermal expansion can be associated to the spontaneous exchange magnetostriction within the uranium sub-lattice.

**Figure 3:**
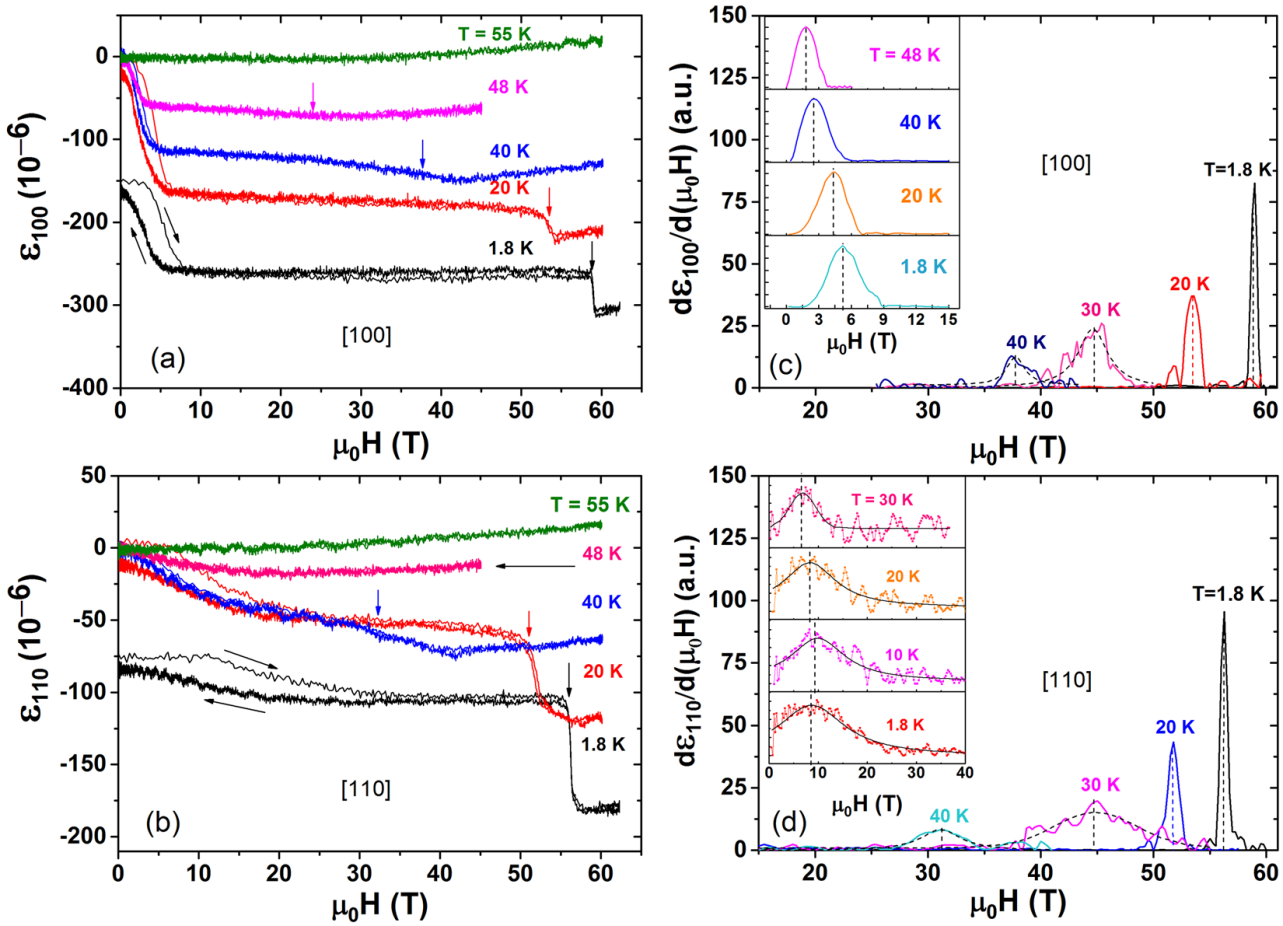
(**a**,**b**) Selected MS isotherms of UN measured along [100] and [110] crystallographic directions. For clarity, the curves measured at T = 1.8 K and along [100] and [110] are shifted vertically by −150 and −75, respectively. The hysteresis between up and down field sweeps of low field transition at T = 1.8 K is shown by the black arrows. (**c**,**d**) The derivatives of MS curves used to determine the values of field at the transitions. The insets show the derivatives of MS at low field range.

In order to investigate the lattice response of UN in external magnetic fields, we have measured MS in pulsed magnetic fields up to 65 T, and temperatures as low as 1.8 K. Figure 3 shows the field dependence of the selected MS isotherms measured along the [100] and [110] crystallographic directions with magnetic fields up to 65 T applied in the same directions of the measurements. In the paramagnetic region (T = 55 K), MS is positive and weakly depends on the magnetic field. For *T* < *T*_*N*_, MS is large and negative, and shows two transitions, one at low field of about 10 T, *H*_*C*1_ and another at high field of about 50 T, *H*_*C*2_. Above the *H*_*C*2_ transition, the magnetostriction is positive. The high field transition at *H*_*C*2_ is sharp at low temperatures (see the MS curve at T = 1.8 K) and turns to a broader transition with increase in temperature. *H*_*C*1_, however, still remains relatively sharp and clear even at temperatures close to *T*_*N*_ (see for comparison the MS curve at T = 48 K). The value of transition fields, *H*_*C*1_ and *H*_*C*2_ are determined by taking the first derivative of the MS with respect to the magnetic field. Figure 3(c) and (d) show the first derivatives of the MS curves. The derivatives in the low field region are shown in the insets. As seen, the derivative at low temperatures gives a very sharp peak that shifts gradually to lower fields at higher temperatures. At a given temperature, *H*_*C*2_ along [110] is similar to that along [100] (see Fig. 3). However, the MS behaviour and *H*_*C*1_ values measured along [110] are quite different from those measured along [100]. In addition, MS along [110] shows a more shallow and broader transition than along [100]. Recent, transport measurements done by Samsel-Czekała *et al*.^6^ show negative anisotropic behaviour of magnetoresistance when measured in different crystallographic directions with similar value of *H*_*C*1_. Moreover, the magnetoresistivity drop is also lower for [110] than [100], as observed in our MS data. The low field transition is characterized by a hysteresis in magnetostriction (see for instance the MS curve at T = 1.8 K in Fig. 3(a)). A similar change in magnetostriction and magnetoresistivity might suggest that magnetic domains play a role at this field range. Because UN has antiferromagnetic order of type-I, there are 3-kinds of equivalent magnetic domains along [100], [010] and [001] directions^10, 25, 32^. The magnetic domains rearrange and align along the direction of magnetic field, which can cause the MS effect in the sample together with the change of the electrical resistivity. Furthermore, the presence of hysteresis between up and down field sweeps in the low field transition might further support the domain origin of large negative magnetostriction and *H*_*C*1_. A comparison of thermal expansion measured at zero and applied fields are shown in Fig. 2. The circle (red), up triangle (green) and down triangle (purple) show $\frac{{\rm{\Delta }}L}{L}(T)$ values obtained at 10, 30, and 60 T, respectively. The dotted lines are guides to the eye that represent how the UN lattice behaves at different temperatures in the presence of magnetic field. Similar to the 0 T results, the thermal expansion under magnetic field is also positive. However, it has different temperature dependence. Furthermore, as marked by the blue arrows, the anomalies around 15 and 42 K seem to also be present in applied magnetic field, especially the one at 42 K.

**Figure 4:**
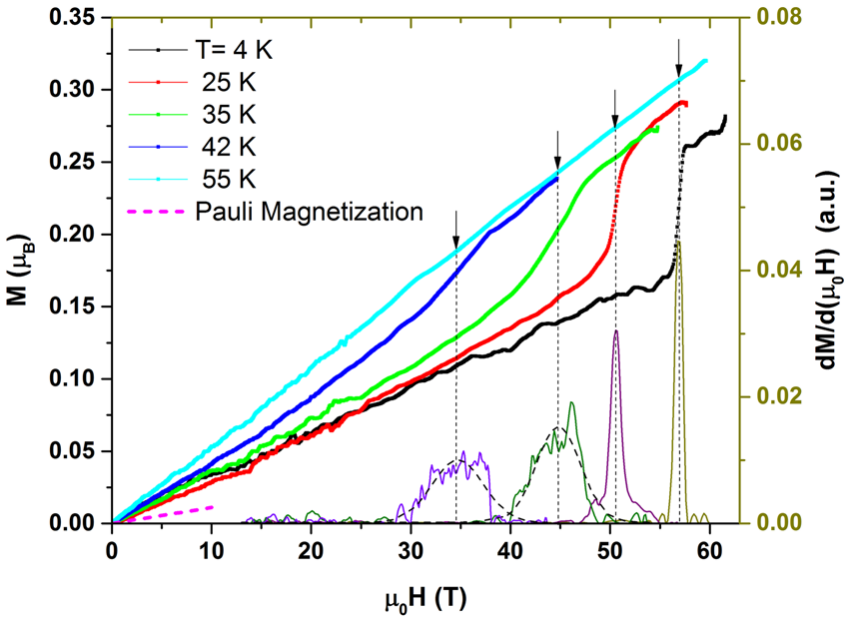
The high field magnetisation of UN single crystal measured at different temperatures. The right hand scale shows the first derivative of magnetisation curve, used to determine the magnetic field value of the metamagnetic phase transitions (shown by the black arrows). The dashed magenta curve shows the estimated Pauli spin magnetisation (see text). The dashed curves are the Lorentzian fits.

In order to further explore impact of the low and high field transitions observed in magnetostriction on magnetism in UN, we have performed magnetisation measurements using a pick-up coil technique (see Method Section). Figure 4 shows the temperature dependence of the magnetisation of UN expressed in Bohr magneton (*μ*_*B*_) in pulsed fields up to 60 T. At T = 55 K (paramagnetic region) the magnetisation shows linear dependence with the applied field without any sign of saturation, even at 60 T. For *T* < *T*_*N*_ the magnetisation curve shows that the metamagnetic transition becomes sharper as the temperature is reduced. *H*_*C*2_ is determined by taking the first derivative, dM/dH, of magnetisation isotherms (right hand scale in Fig. 4). As seen the dM/dH curves show a sharp peak at 57 T for T = 4 K that shifts to the lower fields as temperature grows. Similar values of *H*_*C*2_ are obtained from M(H) and $\frac{{\rm{\Delta }}L}{L}(T)$. The induced magnetic moment above the transition is ~0.25 *μ*_*B*_ at T = 4 K. It is worth noting that this value of the magnetic moment is only 1/3 of the moment obtained by the neutron scattering result, 0.75 *μ*_*B*_^25^. This might suggest only a partial alignment of spins along the field direction, even at 60 T. This result is consistent with a recent high field magnetisation study^19^. Using a layer Ising spins model, it has been predicted that the complete alignment of magnetic moments would be observed at ∼260 T^19^. However, the later estimates neglects the importance of the lattice and its coupling to the spin sub-system. In fact, as shown here, spin-lattice interactions play a crucial role in magneto-elastic properties of UN. Taking into account the density of states at the Fermi level in UN (obtained from the low temperature specific heat, *γ*_*el*_ = 45 mJ/mol K^2^) and using the free electron Fermi gas model we estimated the Pauli-type magnetisation as shown by dashed line in Fig. 4. As seen, the estimated magnetisation is comparable to the measured magnetisation of UN at low fields. This might support an itinerant behavior of 5 *f* electrons in UN, however different experiments show different degree of 5 *f*-elecron localisation in this material (see Refs 6, 19, 25, 33). Clearly, more studies are needed to fully account for this issue in UN.

The application of a magnetic field to an antiferromagnet can cause abrupt changes to its magnetic state. At high magnetic fields, the spins in AFM ordering are forced to align in the direction of magnetic field and this sudden increase of magnetisation is called a metamagnetic transition^34^. There are two types of metamagnetic transitions. When applying a magnetic field parallel to the magnetisation direction it tends to rotate the magnetisation perpendicular to the applied field, that is perpendicular to the easy magnetisation direction. At a critical magnetic field the two sub-lattice magnetisation rotates suddenly to a direction perpendicular to the easy magnetisation direction, consequently perpendicular to the applied magnetic field. This process is called a spin-flop transition and it is characteristic for system with weak anisotropy energy. After that a continuous rotation of the magnetic moment occurs upon increasing field. On the other hand, for systems with large magnetocrystalline anisotropy the magnetisation of the two sub-lattices remains parallel to the easy magnetisation axis up to a critical field. At *H* = *H*_*c*_ a sudden rotation occurs of the sub lattice magnetisation antiparallel to H, towards the field direction resulting to a parallel arrangements of both magnetic moments. The saturation state is obtained, after the transition. This is the so-called spin-flip transition. The overall nature of the transition in UN (from both magnetostriction and magnetisation experiments) suggests that the *H*_*C*2_ anomaly at high magnetic field is, therefore, due to a metamagnetic transition, most probably of the spin-flip type^34, 35^. By looking at the magnetostriction and magnetisation data in the vicinity of *H*_*C*2_ in more detail it seems that a change of a type of the transition from first type at low temperatures and high fields to second order in high temperature and low fields occurs. In general, it is important to locate the phase boundaries and the tricritical point (*T*_*tri*_) as accurately as possible. The first-order line is usually difficult to determine from the magnetisation measurements^34^. The first-order phase boundaries are evidenced and determined by the changes of slope in M as a function of H. However, at *T*_*tri*_ the discontinuity in M shrinks and the susceptibility at the phase boundaries diverges and this makes the anomalies very difficult to locate, especially near the tricritical point. In addition, the susceptibility diverges at the second-order line and the kinks are never infinitely sharp because of factors such as inhomogeneous demagnetizing fields and sample imperfection and quality. Taking this into account we have decided to use high field magnetostriction as a better probe of a tricritical point in UN.

**Figure 5:**
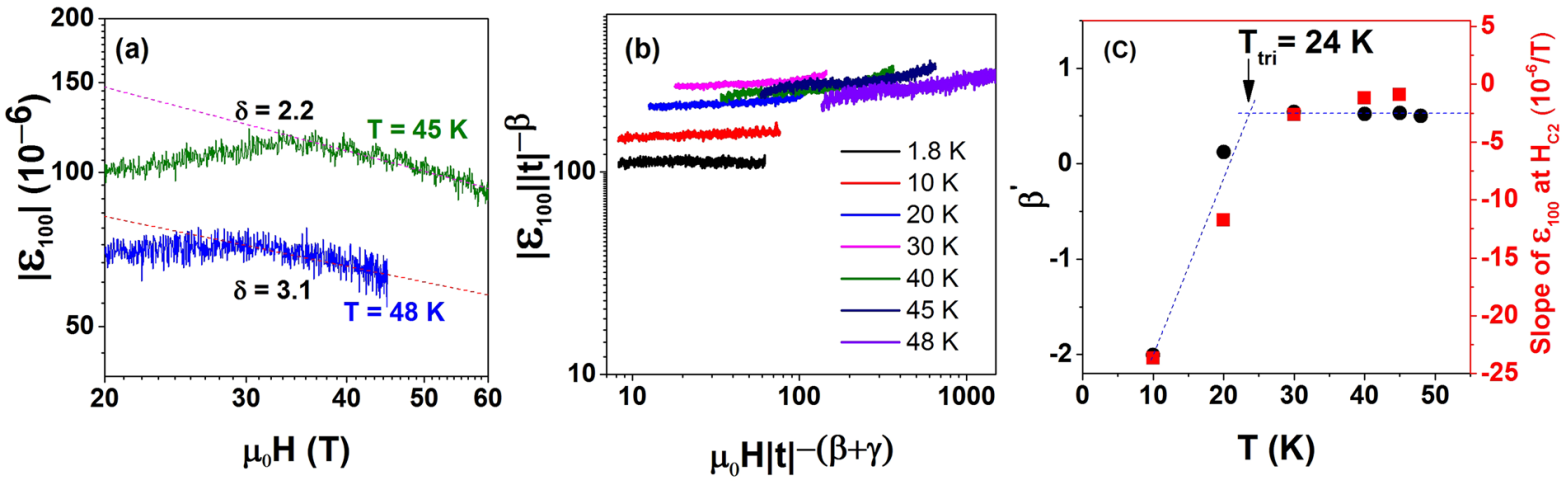
(**a**) Magnetostriction vs. magnetic field of UN at *T* = 45 and 48 K. The dotted lines show a power law behavior (see the text). (**b**) MS scaling curves with critical exponents *β* = 0.5 and *γ* = 1 at different temperatures. (**c**) Temperature dependence of *β*′ (left hand scale, black circles) and slope of MS curves at H_*c*2_ (right hand scale, red squares). The tricritical point at T_*tri*_ = 24 K is determined by the crossing of blue dotted lines (see the text).

Consequently, in Fig. 5(a) we show a |ε_100_| versus *μ*_0_ *H* log-log plot of UN crystal in the high field range and close to antiferromagnetic phase transition. The MS at 48 and 45 K shows a critical power law behaviour *ε*_100_ ∝ H^−1/*δ*^, where *δ* is a critical exponent^36^. The dotted lines shows the fitting of the data to the power law with the critical exponent *δ* = 3.1 obtained for 48 K. This *δ* value is in good agreement with the theoretical value of 3 estimated in the mean field approximation^36, 37^. With lowering temperature the *δ* value decreases and at *T* = 45 K equals to 2.2 (see Fig. 5 (a)). At T = 48 K by using the Widom scaling relation, δ = 1 + *γ*/*β*, and by taking the mean field value of *γ* = 1 we have estimated *β* = 0.48^38, 39^. This value of *β* is close to the mean field result *β* = 0.5^37^. Figure 5(b) shows the plot of |*ε*_100_||*t*|^−*β*^ as a function of *μ*_0_ *H*|*t*|^−(*β*+*γ)*^, where *t* = (*T* − *T*_*N*_) is the reduced temperature^37^ and by taking *γ* = 1 and *β* = 0.5. As seen from the figure the MS curves below *T*_*N*_ and down to 30 K follow the universal scaling law. However, it starts to deviate below T = 20 K. Taking *γ* = 1, we have rescaled the *β* exponent (referred to as *β*′ from now on) such that all the curves follow the universal scaling law. The results are plotted in Fig. 5(c) presenting the so-obtained *β*′ exponent as a function of temperature. In addition, in the same figure we also plot the slope of the MS curves at *H*_*C*2_ (right hand scale of Fig. 5(c)), which has been also proposed as a measure of the type of the phase transition^21^. As seen from the figure, the critical exponent and the slope of the MS start to show anomalous behavior below 30 K. We have defined the tricritical point by fitting the data below and above the transition and then establish its value at the point where these two lines intersect (see dotted lines in Fig. 5(c). As presented in the figure, it gives *T*_*tri*_ ∼ 24 K.

**Figure 6:**
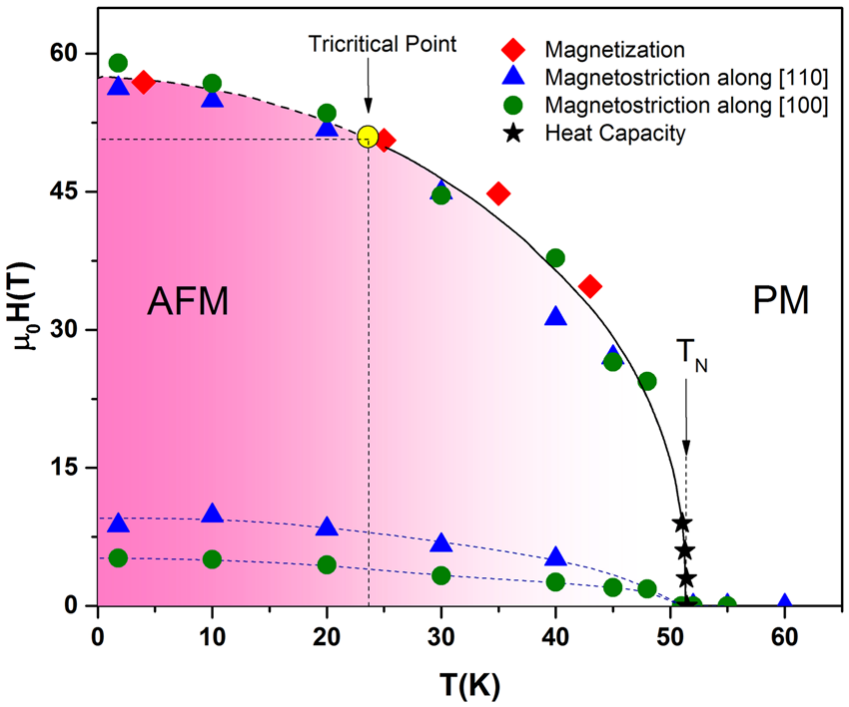
The proposed magnetic phase diagram of UN. The diamonds and stars show the data from magnetisation and heat capacity measurements, respectively. The magnetostriction data measured along the [110] and [100] directions are represented by up-triangles and solid circles, respectively. A solid yellow circle marks the tricritical point at *T*_*tri*_ ∼ 24 K and *H*_*tri*_ ∼ 52 T. The dashed and solid lines mark lines of first and second order transition, respectively and serve as a guide to the eye.

As shown in Fig. 6, by using the magnetostriction, magnetisation, and heat capacity data we have constructed a magnetic phase diagram of UN. The values of *H*_*C*2_ obtained along [100] and [110] are consistent with magnetostriction, magnetisation, and specific heat results. The value of *H*_*C*2_ at 2 K is ∼56 T, it gradually decreases with temperature and reaches ∼30 T at 45 K. In the phase diagram we have also included *H*_*C*1_ values. The tricritical point at *T*_*tri*_ ∼ 24 K and *H*_*tri*_ ∼ 52 T is marked by the yellow circle.

## Summary and Conclusions

We have completed high-field magnetostriction, magnetisation, and heat capacity studies on oriented UN single crystals. The results obtained provide evidence of strong coupling between the magnetism and lattice in this material, which has to be taken into account when analyzing its thermodynamic properties. For both measured crystallographic orientations the magnetostriction is positive and small in the paramagnetic state. Below the Néel temperature the magnetostriction is large and negative and shows a metamagnetic transition at high fields. Our data suggest a change in the nature of the transition from first to second order at a tricritical point at *T*_*tri*_ ∼ 24 K and *H*_*tri*_ ∼ 52 T. Above *T*_*N*_ the magnetisation increases linearly with field, and does not show any sign of saturation in the fields up to 60 T. In the magnetic state M(H) curves show a metamagnetic transition matching high field magnetostriction results. Interestingly, the low field anomaly that strongly affects magnetostriction does not show any effect on magnetisation. The induced magnetic moment at the maximum field of 60 T is only a fraction of that obtained from neutron diffraction and might support the idea that only one set of spins have aligned along the field direction^19^. Neutron diffraction experiments at high fields are necessary to further resolve this issue. Using the results obtained we constructed a new magnetic phase diagram of UN. These studies demonstrate that magnetostriction measurements are an effective probe to investigate the magneto-structural coupling in actinide materials.

## Methods

### Specific Heat Measurements

We measured the specific heat of UN single crystal using a thermal relaxation method in a commercially available Quantum Design, Physical Properties Measurement System (PPMS).

### Thermal Expansion and Magnetostriction Experiments

Thermal expansion and longitudinal magnetostriction measurements on UN crystals in pulsed magnetic fields up to 65 T were carried out using the fiber Bragg grating (FBG) technique, adapted from Daou *et al*.^27^, at the National High magnetic field laboratory (NHMFL), Los Alamos. A UN single crystal of typical dimensions 3 mm × 2 mm × 1 mm size was attached to a 125 μm Telecom single-mode optical fiber, furbished with a 1 mm long FBG. The FBG was illuminated with a broadband light source from a superluminescent diode. Depending upon the sign of strain effect the narrow band of light (≈1550 nm) that is reflected from the FGB shifts slightly. The reflected light is dispersed in a spectrometer and its spectrum is detected by a InGaAs line array camera at 47 kHz.

### Magnetisation Experiments

Magnetisation measurements in pulsed magnetic fields up to 60 T were measured using a pickup-coil technique at NHMFL, Los Alamos. A UN single crystal was enclosed in a capsule and placed directly inside the pickup coils. The measurements were done by applying pulsed magnetic fields when the sample was moved in and out of the pickup coils. The difference in response of the two positions gives the magnetisation of the measures samples. This technique is also known as “extraction magnetometry”.

### Data availability

All data generated in this study are available from the authors upon request.
